# Identifying patients with psychosocial problems in general practice: A scoping review

**DOI:** 10.3389/fmed.2022.1010001

**Published:** 2023-02-08

**Authors:** Rosemarie Schwenker, Tobias Deutsch, Susanne Unverzagt, Thomas Frese

**Affiliations:** ^1^Center for Health Sciences, Institute of General Practice, Martin-Luther-University Halle-Wittenberg, Halle (Saale), Germany; ^2^Department of General Practice, University of Leipzig, Leipzig, Germany

**Keywords:** primary care, general practice, psychosocial problems, social problems, identification tools, scoping review

## Abstract

**Objective:**

We conducted a scoping review with the aim of comprehensively investigating what tools or methods have been examined in general practice research that capture a wide range of psychosocial problems (PSPs) and serve to identify patients and highlight their characteristics.

**Methods:**

We followed the Preferred Reporting Items for Systematic Reviews and Meta-Analyses extension for scoping reviews and the *Joanna Briggs Institute Reviewer’s Manual* on scoping reviews. A systematic search was conducted in four electronic databases (Medline [Ovid], Web of Science Core Collection, PsycInfo, Cochrane Library) for quantitative and qualitative studies in English, Spanish, French, and German with no time limit. The protocol was registered with Open Science Framework and published in BMJ Open.

**Results:**

Of the 839 articles identified, 66 met the criteria for study eligibility, from which 61 instruments were identified. The publications were from 18 different countries, with most studies employing an observational design and including mostly adult patients. Among all instruments, 22 were reported as validated, which we present in this paper. Overall, quality criteria were reported differently, with studies generally providing little detail. Most of the instruments were used as paper and pencil questionnaires. We found considerable heterogeneity in the theoretical conceptualisation, definition, and measurement of PSPs, ranging from psychiatric case findings to specific social problems.

**Discussion and conclusion:**

This review presents a number of tools and methods that have been studied and used in general practice research. Adapted and tailored to local circumstances, practice populations, and needs, they could be useful for identifying patients with PSPs in daily GP practice; however, this requires further research. Given the heterogeneity of studies and instruments, future research efforts should include both a more structured evaluation of instruments and the incorporation of consensus methods to move forward from instrument research to actual use in daily practice.

## 1. Introduction

Since general practitioners (GPs) are usually the first point of contact for people with any health-related concern, patients visit their GP not only for medical reasons but also for psychosocial problems (PSPs) (1–4). Here we take problems to be “a source of perplexity, distress, or vexation”, while we take PSPs to refer to problems related to the conditions in which people are born, grow, live, work, and age (5). These conditions in which people lead their lives have a profound impact on health.

The relationship between these conditions and health has been investigated in numerous studies and addressed in many health reports (6–9). In a general sense, people with PSPs are vulnerable to negative health outcomes, comorbidities, and have a generally poorer health status (10), while PSPs are also related to several more specific conditions, such as cardiovascular diseases, diabetes, infectious diseases, and psychiatric disorders (11–17). This is because PSPs affect immunological and inflammatory processes (18–20) and are associated with an increased risk of illness, delayed recovery, chronic disease progression, and compromised quality of life and mortality rates (10, 21–23). As one example, individuals who are socially isolated are at risk of premature mortality, comparable to well-documented risk factors, such as smoking and obesity (11, 24–27). Furthermore, psychosocial factors at work have been shown to be associated with a range of health outcomes, such as job strain and increased risk for heart disease or diabetes (28–31). Similarly, job insecurity and unemployment have been found to be associated with an increased risk for cardiovascular and coronary heart disease (32).

The issue of PSPs in general practice began to draw greater attention in the 1990s (33–35) and a vast body of research has investigated their significance since then. For instance, studies show that at least one third of patients under general practice report experiencing PSPs and that GPs are consulted by patients with PSPs at least three times per week (3, 33, 36). Studies most frequently identified family problems, caregiving tasks, violence-related issues, isolation, financial problems, employment problems, problems with physical functioning, and legal problems (3, 35, 37–46). The most frequently occurring social problems encountered in the primary care context are captured in the International Classification of Primary Care (ICPC-2) (47). However, studies also show that GPs only correctly identify a fifth to a half of patients with relevant PSPs and that social factors are not considered to have much importance (37, 48). Possible consequences, such as inadequate diagnoses, non-specific or no intervention or treatment at all, and ineffective use of time have also been extensively described (2, 34, 35, 37, 40, 41, 43, 44, 49–55).

The complexity and heterogeneity around PSPs leads to difficulties in providing or referring to a universally valid concept. This is compounded by the fact that several disciplines, organisations outside the academic context, and policy makers use different concepts, which in turn leads to difficulties in developing or using a practical approach in the form of a systematic and structured instrument to identify patients with PSPs. This is reflected in the official guidelines of medical organisations or societies and official health organisations, where the integration of the psychosocial perspective into medicine is widely demanded, but concrete steps for practice are still lacking (3, 10, 55–58).

GPs are in a unique position to take preventive action to promote health and to identify and treat emerging health problems early in routine care. The use of tools to identify patients with PSPs could be useful in this regard. With this in mind, the aim of our study is to provide an overview of the published research in which we present what tools or methods have been studied so far in general practice research that capture a wide range of PSPs and could be used to identify patients presenting with PSPs.

## 2. Methods

We conducted a scoping review by following the *Joanna Briggs Institute Reviewer’s Manual* (59) and the Preferred Reporting Items for Systematic Reviews and Meta-Analyses extension for Scoping Reviews checklist (60). A protocol has been registered with Open Science Framework (OSF) (61) and published in BMJ Open (62). Since the entire review process with its individual phases is iterative and fundamentally a process of developing understanding, hermeneutic principles were applied consisting of two mutually influencing cycles; searching and accessing the literature, and analysis and interpretation (63).

The review process was conducted in an intersubjective manner as part of the collaborative research process. Collaboration provides a check and balance through which an analytical consensus can be reached that allows for a more comprehensive interpretation and for the group analysis to move beyond individually preconceived perspectives. Through the process of multiple researchers articulating, clarifying, and challenging their initial interpretations, consensus was reached on which studies should be included, which represented the relevant information for extraction, and how they should be summarised and classified (64).

### 2.1. Inclusion criteria

Evidence sources were considered for inclusion if they met the criteria specified by the JBI based on the population, concept, and context framework (59) (Table 1). Included evidence sources were required

**TABLE 1:** Eligibility criteria based on study population, context, concept, and types of sources of evidence.

	Inclusion criteria	Exclusion criteria
**Population**	• Adolescents, adults, or elderly people	• Adolescents, adults, or elderly people with disease-specific PSPs related to, e.g., cancer, HIV, diabetes, substance use disorder, or psychiatric disorders
**Concept**	• Any kind of instrument or approach (e.g., questionnaire, interview) to identify patients with PSPs in general or regarding specific social problems according to ICPC-2, Section Z^2^, and any kind of reporting format (e.g., self-reported, physicians’ assessment)	• Identification instruments or approaches based on the report of patients’ parents, carers, or other significant others
**Context**	• General practice settings • Any geographical location	
**Types of sources of evidence**	• Peer-reviewed publications • Primary empirical research studies (e.g., quantitative, qualitative, or mixed-methods studies) • Theoretical articles describing an instrument used for identifying patients with PSPs in sufficient detail • Full-text publications according to the EQUATOR Network (60) guidelines • Articles written in English, Spanish, French, or German, without time restriction	• Editorial articles (e.g., perspective pieces, opinion papers, position statements) • Study protocols • Abstracts and posters • Author replies/comments • Dissertations/theses • Articles for which we could not obtain the full text or that are not written in English, Spanish, French, or German
EQUATOR, Enhancing the QUAlity and Transparency Of health Research; PSPs, psychosocial problems		

^2^Poverty/financial problem (Z01), Food/water problem (Z02), Housing/neighbourhood problem (Z03), Social cultural problem (Z04), Work problem (Z05), Unemployment problem (Z06), Education problem (Z07), Social welfare problem (Z08), Legal problem (Z09), Health care system problem (Z10), Compliance/being ill problem (Z11), Relationship problem with partner (Z12), Partner’s behaviour problem (Z13), Partner illness problem (Z14), Loss/death of partner problem (Z15), Relationship problem with child (Z16), Illness problem with child.

(1) to refer to the adolescent, adult, or elderly population in general practice settings and
(2) to describe any kind of tool or approach to identify patients with PSPs.

We took into consideration articles that included PSPs in general, as well as articles that focused on specific social problems according to the ICPC-2. As the term “psychosocial problems” is used very differently and inconsistently in different publications and even within the same publication, no strict definition was set as an inclusion criterion and therefore we also included publications that refer to PSPs but were labelled as, for example, “mental health problems,” “psychological distress,” or “emotional stress.” This approach was taken to ensure that the descriptions in the articles identified in the search could use any definition of PSPs and still be included. However, we wanted the “social” aspect to be present and were particularly interested in tools that focus on assessing patients’ problems rather than making a formal diagnosis. This process was carried out through an independent review of the full texts by two reviewers and consensus building when conflicts arose.

In line with the characteristics of a scoping review, all types of empirical research studies were included (59). Not all articles reported studies. In those cases where no study was reported, but an instrument or approach for identifying patients with PSPs was described in sufficient detail, the articles were included. The search was limited to references published in English, Spanish, French, and German, without time restriction, and from any geographical location. We excluded articles in which the population described consisted of patients with PSPs related to specific chronic diseases or conditions (e.g., cancer, HIV, diabetes, substance use, or psychiatric disorders) as there are specific research areas for these and their inclusion would have been beyond the scope of our study and inconsistent with our focus on the general population. We also excluded studies where the identification tools or approaches were based on reports from third parties, (e.g., parents), rather than from the participants themselves or a healthcare professional.

### 2.2. Search strategy

A preliminary search was conducted in MEDLINE (Ovid) database to gain familiarity and an overview and to identify key terms. We developed a search strategy for MEDLINE (Ovid) (see Supplementary Table 1) and adapted this strategy to the databases PsycInfo, the Cochrane Library, and the Web of Science Core Collection. The search took place from March to April 2021. We hand-searched and screened the reference lists of the included evidence sources to identify other potential references. We screened the reference lists of systematic reviews and scoping reviews which examined studies potentially fitting our inclusion criteria for further relevant articles. Search results were exported into EndNote 20. After elimination of duplicates, the remaining references were uploaded to Covidence for screening and data extraction. An updated search was performed in June 2022.

### 2.3. Source of evidence screening and selection

Two reviewers (RS, TD) independently screened the references by title and abstract. The full texts of selected articles were then retrieved and fully read by the same two reviewers. In both steps, discrepancies between reviewers’ assessments were discussed and solved by consensus. A list of included studies is presented in Supplementary Table 2.

### 2.4. Data extraction

A data extraction template was devised by the primary author (RS) to capture information relevant to the research question (see Supplementary Table 3). Two reviewers (RS, TD) independently performed a pilot data extraction on a random sample of five articles and subsequently refined the form. Data extraction was conducted independently by RS and a study assistant who met frequently with TD to discuss the process and refine the data extraction form to ensure that all information relevant to answering the research question was extracted from the publications. Reviewers extracted findings as reported, in the form of numerical or narrative summary statements. Disagreements were resolved through consensus building and, if necessary, by involving a third reviewer.

### 2.5. Data synthesis and analysis

Following data extraction, each study was categorised according to year of publication, country, setting, research design, population, and the tool(s) or method(s) described (see Supplementary Table 4). A narrative of the data was then developed.

### 2.6. Deviations from the protocol

As there are not only studies but also publications that describe schemes, frameworks, or instruments to theoretically identify patients with PSPs, we decided to refine our inclusion criteria accordingly. For articles reporting Randomised Controlled Trials (RCTs), we decided to include not only identification tools, which were mostly used to recruit study participants, but also outcome assessment tools if they met our inclusion criteria. The original data extraction form proposed in the protocol was modified during the pilot data extraction phase in order to capture the most relevant aspects of the included articles.

## 3. Results

### 3.1. Study selection

**FIGURE 1:**
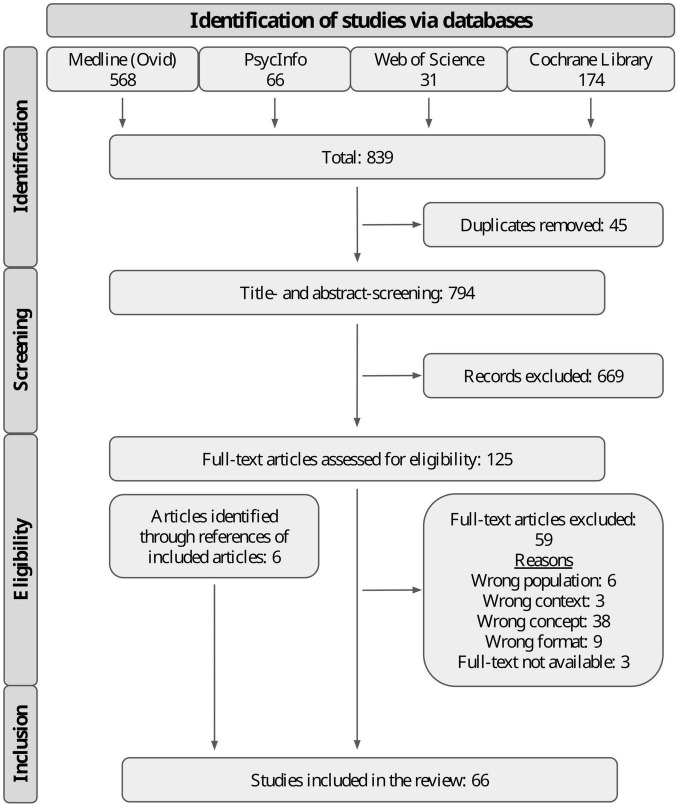
PRISMA flow diagram.

The searches of electronic databases resulted in 839 records. After removing duplicates, a total of 794 titles and abstracts were screened, from which 669 articles were removed, with 125 articles then subjected to a full-text review. In this step, a further 6 additional records were identified through references from identified articles. Of the full-text articles reviewed, 59 were excluded. This left 66 studies that were considered eligible for inclusion and from which relevant information was extracted. Figure 1 presents the PRISMA flow diagram. Reasons for exclusion are reported in Supplementary Table 5.

### 3.2. Study characteristics

Supplementary Table 4 presents the general study characteristics of the 66 studies included in this review. The articles included were published between 1978 and 2020. We identified a total of 61 instruments. In this paper, we present only validated instruments (22). Results for the remaining instruments without validation will be published separately.

#### 3.2.1. Context (country and setting)

We identified publications from 18 countries, with the most deriving from the United Kingdom (13), the Netherlands (9), the United States (7), Australia (7), and New Zealand (5). There is a clear concentration of studies conducted in Europe (39), followed by Australasia (12), and North America (9). Of the 66 included studies, 64 come from high-income countries, with 1 article each from an upper-middle income country (Brazil) and a lower-middle income country (Pakistan).

All publications referred to a general practice setting. However, our study allowed for different regional uses of terms. This means that the studied context is not only referred to as general practice (41), but also as primary care (15) and family medicine (8), while the terms general internal medicine (1) and health care centre (1) were also used.

#### 3.2.2. Research design

Most of the studies included were analytical (62). Among these, observational studies were the most common (42), such as cross-sectional studies and prospective studies, followed by experimental studies (12), such as (cluster) randomised controlled trials (RCTs). Descriptive studies were also included, such as narrative/literature reviews (4). No qualitative studies were identified.

#### 3.2.3. Population

Publications included in this review focused mostly on adults (49), with others focused on elderly people (10) and on adolescents and/or young people (7). Specific characteristics among the adult population refer to pregnant women (1), veterans (1), caregivers of patients with a chronic condition (1), and people classified as lesbian, gay, or bisexual (1). Almost half of the studies included patients (31), with others focused on caregivers (1) and citizens (2), while the other half worked with patients and physicians and/or practice nurses/staff, and/or community mental health nurses (31). One article focused only on physicians. Study samples show a higher proportion of those identified as of biologically female sex.

### 3.3. Instrument characteristics

Of the 66 articles, 48 used 1 instrument, 8 publications used 2 instruments, and 10 articles used 3 or more instruments that met our inclusion criteria. Table 2 shows the characteristics of the instruments in terms of instrument name, study, stated focus, and age group. Table 3 contains further aspects, such as screening setting, type of instrument, screening method, mode of administration, regularity of screening, and whether and what information on quality criteria were assessed. In total, 61 instruments or methods were identified. Of these, 22 were reported as validated and are presented below.

**TABLE 2:** Instrument characteristics.

Instrument	Study	Focus of instrument reported in the study	Age group
**Thematic pattern I: Comprehensive (medical, psychological and/or psychosocial and/or social aspects)**			
Adapted General Well-Being Index (AGWBI)*	Hopton (87)	Experience of illness, distress and psychosocial difficulties: anxiety, depression, positive wellbeing, general health perceptions, vitality, and perceptions of self-control; common worries or difficulties: relationship, partner, money, housing, work, social activities, unemployment, children, family members, violence/threat of violence, death of a close person	Adults
Case-finding and Help Assessment Tool (CHAT)*	Goodyear-Smith (88, 68)	Lifestyle and mental health assessment: tobacco use, alcohol and other drug misuse, problem gambling, depression, anxiety and stress, abuse, anger problems, inactivity, eating disorders, insomnia	Adults
Coding System for Primary Health Care (CPHC)*	Deliège (89)	Social problems and complaints: family problems, social integration problems, socio-economic problems and basic needs, problems of social status and occupation, problems with social institutions, problems of violence in society, functional and social consequences of diseases, other problems	Adults
Electronic Case-finding and Help Assessment Tool (eCHAT)	Goodyear-Smith (90)	Lifestyle risk factors: smoking, problematic drinking, other drug use, gambling, exposure to abuse, physical inactivity; mental health issues: depression, anxiety, anger control	Adults
Multifactor Health Inventory (MHI)	Hase and Luger (91)	Symptoms and problems: physical symptoms and possible psychological and behavioural problems, attitudes	Adults
Observation List for Mental Disorders and Social Problems (OLP)	Tak (92)	Mental disorders and social problems: anxiety, depression, cognitive impairment, suspicion, loneliness, somatisation	Elderly people
Personal Inventory	Hilliard (93)	Psychological functioning: quality of intimate relationships, emotional distress, concerns about employment and finances, personal energy, coping	Adults
Short Form Health Survey (SF-12)*	King (94)/Schreuders (95)/MacMillan (67)/Freund and Lous (65)/Hegarty (96)/Geyti (79, 97)	Physical and psychological well-being/Global mental and physical health and well-being/Health-related quality of life/Mental health status/General health, mental health, and health behaviour, mood and anxiety symptoms, physical health, functional limitations/Mental health status, health-related quality of life	Adults
Short Form-36 (SF-36)*	Raine (98)/Schreuders (95)/Hassink-Franke (99)	Social functioning, mental health, role limitation due to emotional problems, general health perception/Physical functioning, role limitations due to mental health problems, bodily pain, general health perceptions, vitality, social functioning, role limitations due to emotional problems and general mental health/Physical and mental health	Adults
**Thematic pattern II: One or more social problem(s)**			
Composite Abuse Scale (CAS)*	MacMillan (67)/Sanci (100)	Intimate partner violence and physical, sexual, and emotional abuse in the last 12 months	Adults
Family APGAR*	Hilliard (93)/De la Revilla (101)	Family functioning: adaptation, partnership, growth, affection, and resolve	Adults
Family Strain Questionnaire Short Form (FSQ-SF)*	Vidotto (102)	Emotional burden, problems in social involvement, need for knowledge about the disease, satisfaction with family relationships, thoughts about death	Adults
Hopkins-Symptom Checklist (HSCL-25)	Stefansson and Svensson (103)	Social problems: work, financial, childcare, housing, social isolation	Adults
Patient Health Survey	Wasson (104)	Abusive relationships/Domestic abuse: problems in the household that led to insulting/swearing, yelling, threatening, hitting/pushing	Adults
Social Needs Checklist (SNC)	Cook (105)	Social and environmental problems: finances, personal stress, family problems, legal, employment or career issue, transportation, too much time alone, activities of daily living, shopping, laundry or house cleaning, other needs, cooking, home health care, housing, obtaining nursing home placement	Adults
Social Problem Questionnaire (SPQ)	Al-Shammari (106)/Saltini (107)	Social problems: housing, work, finances, social activities, marital, children, other domestic relationship, relationship with others (e.g., extended family)/Social problems (financial, housing, occupational, social/marital relationship)	Adults
**Thematic pattern III: Psychological aspects**			
Four-Dimensional Symptom Questionnaire (4DSQ)*	Terluin (108)	Common psychological symptoms, distinguish non-specific general distress from depression, anxiety and somatisation; distress: worry, irritability, tension, listlessness, poor concentration, sleeping problems, demoralisation	Adults
General Health Questionnaire (GHQ-12) *	Van der Pasch and Verhaak (109)/Kapur (110)/Saltini (107)/Kendrick (111)/Mirza (112)/King (94)/Schreuders (95)/Goncalves (113)/Hassink-Franke (99)	Psychosocial complaints (common mental disorders); reason for the visit, onset of their problem, name of problem, problem severity, what to fear most about problem, activities and functioning (difficulties), affect on body parts/Psychological distress/Mental health problems/Common mental disorders/Emotional distress/Current mental health problems/Emotional symptoms	Adults
General Health Questionnaire (GHQ-28) *	Shiber (114)/De la Revilla (101)/Raine (98)/Watts (115)/De la Revilla Ahumada (116)/Rabinowitz (117)	Emotional problems/Psychosocial problems: somatic symptoms of psychological origin, distress/anxiety, social dysfunction and depression/Mental health problems/Psychosocial problems: somatic symptoms, distress/anxiety, social dysfunction and depression/Psychological distress: somatic symptoms (e.g., feeling run-down), anxiety/insomnia (e.g., lost sleep over worry), social dysfunction (e.g., taking longer over things), and severe depression (e.g., life not worth living)/Psychological problems	Adults; Elderly people
General Health Questionnaire (GHQ-30)	Verhaak (118, 119)/Odell (120)/Smith (66)	Physical, psychological, social problems, emotional distress/Psychiatric morbidity/Mental disorders	Adults
General Health Questionnaire (GHQ-60)	Corser (121)	Emotional state, psychological complaints	Adults
Strengths and Difficulties Questionnaire (SDQ)*	Martinez (122)	Psychological difficulties: overall distress, social impairment, burden and chronicity of the problem	Adolescents

Q, Questionnaire; I, Interview; M, Mnemonic; *Freely available.

**TABLE 3:** Instrument characteristics in detail.

Instrument	Screening setting	Type of instrument	Screening method	Administered by	Regularity of screening	Information on		
						Validity	Reliability	Feasibility
**Thematic pattern I: Comprehensive (medical, psychological and/or psychosocial and/or social aspects)**								
Adapted General Well-Being Index (AGWBI)*	NR	Q	Paper and pencil	Patient	NR	Assessed in the study	Yes	NR
Case-finding and Help Assessment Tool (CHAT)*	In the waiting room, prior to consultation	Q	Paper and pencil	Patient	NR	Assessed in the study	Yes/NR	2 min. to complete
Coding System for Primary Health Care (CPHC)*	NR	Classification system, conceptual framework	NR	Physician	NR	Yes	Yes	NR
Electronic Case-finding and Help Assessment Tool (eCHAT)	In the waiting room, prior to consultation	Q	Computer or tablet	Patient	NR	Yes	NR	Yes
Multifactor Health Inventory (MHI)	In the waiting room, consultation room	Q	Paper and pencil	Patient	NR	External validity	Stability	10 min. to complete
Observation List for Mental Disorders and Social Problems (OLP)	NR	Q	Paper and pencil	Physician, home care worker	After observation in regular visit	Convergent validity	Internal consistency	Few min. to complete
Personal Inventory	NR	Q	Paper and pencil	Patient	Newly registered patients	Yes	Yes	NR
Short Form Health Survey (SF-12)*	NR	Q	Web-based, paper and pencil	Citizen/Patient	Initial health check/outcome assessment in RCT	Yes	NR	NR
Short Form-36 (SF-36)*	NR/prior to consultation	Q	Paper and pencil	Patient	NR/Outcome assessment	Yes	NR/Yes	NR
**Thematic pattern II: One or more social problem(s)**								
Composite Abuse Scale (CAS)*	NR	Q	Paper and pencil	Patient	Screening for study recruitment/Outcome assessment	Yes	NR	NR
Family APGAR*	Prior to consultation	Q	Paper and pencil	Patient	Newly registered patients	Yes	NR/Yes	NR
Family Strain Questionnaire Short Form (FSQ-SF)*	In the waiting room or during consultation	Q, I	Paper and pencil	Patient, physician, other	NR	Convergent, discriminant validity	Internal consistency	5 min. to complete
Hopkins-Symptom Checklist (HSCL-25)	NR	Q	Paper and pencil	Patient	NR	Yes	NR	NR
Patient Health Survey	NR	Q	Paper and pencil	Patient (Women)	NR	Face, criterion validity	Yes	NR
Social Needs Checklist (SNC)	NR	Q	Paper and pencil	Social work students	NR	Face validity	Internal and test-retest reliability	NR
Social Problem Questionnaire (SPQ)	NR	Q	Paper and pencil	Patient, nurse/Patient	Newly registered, unfamiliar, or reticent patients/NR	Yes	NR	5–15 min. to complete
**Thematic pattern III: Psychological aspects**								
Four-Dimensional Symptom Questionnaire (4DSQ)*	NR	Q	Paper and pencil	Patient	NR	Convergent, construct validity	Yes	NR
General Health Questionnaire (GHQ-12)*	NR	Q, I	Paper and pencil	Patient/Patient, physician, other	Initial examination/Screening for study recruitment/Outcome assessment	Yes	NR	NR/Yes
General Health Questionnaire (GHQ-28)*	Prior to consultation/NR	Q	Paper and pencil	Patient/Patient, physician	NR	Yes	NR	NR
General Health Questionnaire (GHQ-30)	Prior to consultation/NR	Q	Paper and pencil	Patient/Patient, physician	NR/Outcome assessment	Yes	NR	Few min. to complete
General Health Questionnaire (GHQ-60)	Prior to consultation	Q	Paper and pencil	Patient	NR	Yes	NR	NR
Strengths and Difficulties Questionnaire (SDQ)*	NR	Q	Paper and pencil	Patient	NR	Yes	Yes	NR

Q, Questionnaire; I, Interview; M, Mnemonic; NR, Not reported; *Freely available.

The instruments used mostly targeted adults (21), among which two targeted elderly people. One instrument targeted adolescents. For all but one instrument, which contained information reported by the physician, participants themselves were the informants. Few instruments included additional information reported by health professionals (7).

The instruments found were deployed in different formats, mostly as questionnaires (19). Two instruments were used in mixed formats; e.g., as a questionnaire combined with an interview. One instrument was described as a classification system and conceptual framework. The instruments were mostly completed with paper and pencil (19) but also on a tablet or computer (2). For one instrument, the method was not reported.

Information on quality criteria, such as validity, reliability, feasibility, and/or other aspects assessed in relation to the use of the tool were reported differently. When reported, validity was reported in different ways, including internal validity, face, criterion, construct, and external validity. Among these, a validity test was conducted in the study we included for two instruments. For 14 instruments, no detailed information was reported.

Reliability was stated for 10 instruments, of which only four had more detailed information described. Reliability referred to internal consistency, stability, and test-retest reliability. For nine instruments, no information on reliability was provided. For three instruments, different information was available depending on the study description: “yes” and “not reported”.

Feasibility was also reported in different ways, generally not systematically. For the majority of instruments (18), no information was provided. For two instruments, it was stated that feasibility was given, but neither had further details provided. For two instruments, different information was available depending on the study: “yes” and “not reported.”

Other aspects were mentioned for the evaluation of the instruments, such as acceptance, accuracy, applicability, comprehensibility, effectiveness, efficiency, and usefulness, but the information provided was neither detailed nor structured. For two instruments, no information was provided at all. Our research revealed that 12 of the 22 instruments are currently freely available, while 10 are not.

For most instruments (13), no information was provided on the screening setting (e.g., at home, in the waiting room). When reported, the instruments were mostly completed in the waiting room before, during, or after the medical consultation (9). Instruments were completed within five to ten or fifteen minutes (6), but for most instruments, completion time was not reported (16).

For most instruments (13), no information was provided on the regularity of screening. When reported, instruments were used with newly registered and/or unknown patients (4). In three cases, the tool was used to recruit participants for the study and/or to assess outcomes. Two instruments were used for both purposes.

Table 2 provides information on the focus of each instrument. In general, various aspects around PSPs are covered, varying to some extent depending on the study’s objective, target population, and setting. We found considerable heterogeneity in the theoretical conceptualisation, definition, and measurement of PSPs, ranging from psychiatric case findings to specific social problems. To ensure accuracy, we reported the focus of the identified instruments individually.

Finally, because we sought to offer insights into the instruments’ reported focus, it is worth mentioning three thematic patterns to which the instruments can be assigned. The majority of instruments (9) have a comprehensive focus that includes medical, psychological and/or psychosocial, and/or social aspects (e.g., geriatric assessment). In addition, we found seven instruments measuring one or more social problems (e.g., related to family, partner violence, finances). When specific social problems were addressed, they were related to intimate partner violence (IPV) in female participants (2). Finally, we also found instruments (6) measuring psychological aspects (e.g., stress, anxiety, depression) to identify probable “cases.”

Not all studies included a conclusion or a conclusion relevant to our research question. Furthermore, there were often no specific conclusions on individual instruments, as these were mostly described in the context of the general study content and objectives as well as other instruments. Where indicated, conclusions related to validity, appropriate classification, identification/detection rates, benefits and improvement in case finding, feasibility, administration, awareness, acceptability, usefulness, a starting point for further discussion and/or subsequent interventions, implementation in daily practice, and also limitations.

In most cases, the authors used a narrative approach to draw conclusions about the instruments studied. These contained statements such as “insight [provided by the questionnaire] made it easier for GPs to offer patient-centred counselling and ask questions that offer a holistic picture” (SF-12) (65), “acceptable addition to the consultation to facilitate emotionally distressed patients” (GHQ-30) (66), “helpful and effective to get an overview of the problem” (CAS) (67), and “simple, efficient, and well-suited to the resource- and time-strapped primary care environment” (CHAT) (68). The benefits of the tools for patients were seen to be stimulating conversations, drawing attention to patients, and giving them the opportunity to voice their concerns. The benefits for physicians were seen to be improving recognition skills, initiating conversations, modelling/structuring the conversation, and gaining new information, especially with new and/or reluctant patients. Concerns/limitations were expressed that PSPs-recognition does not necessarily lead to subsequent intervention.

## 4. Discussion

Our research yielded 66 articles that met our inclusion criteria and revealed 61 instruments that were developed or used in general practice research in the general population over a five-decade period. In this paper, we presented 22 instruments that were reported as validated.

We identified a wide range of instruments, including validated instruments (e.g., General Health Questionnaire, GHQ; Short Form Health Survey, SF-12), mnemonics (e.g., HEEADSSS [Home environment, Education and employment, Eating, peer-related Activities, Drugs, Sexuality, Suicide/depression, Safety from injury and violence] and SHADESS [Strengths, school, Home, Activities, Drugs, Emotions/eating, Sexuality, Safety]), and instruments specifically designed for trial recruitment. Although we only present the validated instruments in this article, our findings show that non-validated instruments account for almost two thirds of all identified instruments. This could be understood to mean that non-validated instruments are considered as useful as validated instruments.

Notably, overall we found a relatively high number of instruments compared to the number of publications, which is understandable due to the broad use of terms related to PSPs, which varied both between and within publications. Our results show that the large number of instruments found cover a broad area, with most having a comprehensive focus that includes medical, psychological, and/or psychosocial, and/or social aspects. We found social and psychosocial aspects included in several instruments, albeit conceived and reported very differently.

In the research literature, we also found that the consideration of social contexts and problems is described using different terms; for example, psychosocial and social problems, health-related social needs or risks, or social determinants of health. The same applies to the content and focus of the instruments. While we focused our search and study selection on problems and risks, there is also essential work that assesses underlying structural aspects that lead to problems. For example, Bourgois and colleagues present a structural vulnerability assessment tool to help physicians go beyond risk behaviours to consider the negative health consequences of poverty, inequality, and discrimination, and identify patients who may benefit from additional health and social services (69).

The influence of social circumstances on health is known and recognised, as is the importance of holistic care concepts that take social factors and needs into account (70, 71). Practical implementation by using tools to identify problems and needs has also gained attention in recent years (72, 73). In light of the COVID-19 pandemic, the complex interplay between health, social circumstances, and PSPs has become even more apparent, and concerns are being raised about the psychosocial consequences and long-term negative effects of the pandemic that clinical practice must be prepared to address in order to provide appropriate care and support (74–76).

Similarly, the studies included in our review show the importance of using tools to raise awareness and provide a basis for discussions about PSPs and the associated health risks. In fact, Webb et al., for example, found that a key benefit of using a tool for young people was that it increased confidence in discussing sensitive issues with their GP (77). In Blom et al.’s study, GPs reported that the tools provided them with additional new information and made them more aware of the functions and needs of older people. However, they reported finding it cumbersome to organise multidisciplinary consultations (78). According to Freund and Lous, the use of the questionnaire (SF-12) can provide insight into the relationship between social life, health, lifestyle, and one’s response to stressors and resources. This insight makes it easier for GPs to provide patient-centered counseling and ask questions that provide a holistic picture of the participant (65). Geyti et al. addressed another aspect in their study, noting that the identification of PSPs does not necessarily lead to the initiation of treatment, where they see a need for further research (79).

Recent studies show that the responsibility for the subsequent resolution of PSPs does not have to lie solely with GPs. Studies from Germany show that GPs both feel responsible for dealing with social problems and are able to manage them themselves. However, the need for external support was also expressed, as was the view that interprofessional cooperation is helpful and necessary (3, 80–82). Collaboration between social work and primary care, for example, is considered beneficial and studies show that subjective health, functioning, and self-management can be improved and psychosocial morbidity and barriers to treatment and health maintenance reduced (83, 84). Therefore, identifying patients with PSPs is a crucial step to ensure that patients receive appropriate care in a timely manner, according to their needs and preferences.

The quality criteria and other aspects described for the evaluation of the instruments identified were different, which makes a comparison difficult and does not allow us to formulate a statement on which instrument(s) can be recommended for use in practice. At the same time, the structural and contextual heterogeneity of the instruments makes prioritisation according to purely diagnostic quality criteria impossible. In the absence of this kind of evidence, an approach that combines available data with the experience and insights of clinical experts from multidisciplinary backgrounds is valuable because it provides guidance where none otherwise exists. Consensus procedures, such as the RAND Corporation/University of California Los Angeles (RAND/UCLA) Appropriateness Method can be helpful in this regard, as they can be used to define appropriate indications and develop criteria for use, care, and management (85).

From our findings and discussion points, we derive the potential for subsequent research and the link to practice. For example, investigations could be undertaken into whether and which instruments are actually used by general practice professionals in daily practice or why not. Since we did not examine the quality criteria and other criteria used to evaluate the instruments in more detail, as this would have been beyond the scope of our work, future studies could address this aspect by carrying out methodological studies to examine validity, reliability, and feasibility in more depth. If this work is continued, we believe that mixed methods should be used; these are considered particularly important in complex fields such as health and social sciences, as they allow researchers to gain a deeper understanding and answer research questions that cannot be answered by quantitative or qualitative methods alone, thus addressing the complexity of real-life challenges (86). In order for the step from research to practice to succeed, a consensus procedure like the one just described would be an important contribution to develop a selection of instruments that could or should be used in practice from the perspective of practising healthcare professionals. The development of a corresponding guideline would also be worth considering.

When trying to synthesise such a complex topic there are certain limitations and potential biases. First, we did not assess the quality of the included articles, as this is not the aim of a scoping review. Referring to the WHO and ICPC-2 definition/framework in our understanding of PSPs may have led us to exclude articles that other definitions would have encompassed. Because our results refer to information extracted from the included articles, where information on validation was not always provided, instruments may have been excluded even though validation might have been available. Since we did not set time limits, we included articles from 1978 to the present. Both the way research is conducted and reported and the way PSPs are addressed have changed over that time, limiting the summary and comparability of studies. We note that our findings are predominantly based on literature from high-income countries. PSPs vary in areas with different social and cultural norms and belief contexts, so the results cannot simply be extrapolated to other countries or communities on other continents.

We believe these limitations, however, are offset by numerous strengths. A clear strength of this scoping review is the integration of a wide variety of studies using tailored search strings providing a comprehensive summary of the field. Additionally, strengths include the use of rigorous scoping review methods and compliance with standards for conducting and reporting reviews. All articles were reviewed and extracted by two independent authors to reduce the risk of selection bias. Regular meetings and discussions within our multidisciplinary team, consisting of a sociologist, psychologist, GP, and a mathematician ensured the integration of interdisciplinary perspectives. We have included all types of study designs, reflecting the fact that RCTs are not always appropriate for reporting on a complex topic, which PSPs undoubtedly are.

The integration of psychosocial and social aspects into clinical practice is receiving increasing attention in medicine. The use of identification instruments could be helpful in daily practice to identify patients with PSPs who may benefit from greater support in one or more areas, thus promoting whole-person care for the entire population. This review identified 66 articles reporting on 22 validated instruments that have been studied and used in general practice research. Although the diversity of terms and instruments makes compiling, discussing, and summarising the literature a challenge, the diversity of instruments also demonstrates the great potential and the many ways and variations in which instruments can be used in clinical practice to achieve a deeper understanding and more appropriate care. Adapted and tailored to local circumstances, practice populations, and needs, they could be useful in daily GP practice; however, this requires further research. Given the heterogeneity of studies and instruments, future research efforts should include both a more structured evaluation of instruments and the incorporation of consensus methods to move forward from instrument research to actual use of instruments in daily practice.

## Author contributions

RS, SU, and TF conceptualised and designed the study. TD made contributions. RS and SU developed the search strategy. RS conducted the literature search, developed the data extraction template, performed data analysis and synthesis, wrote the original draft, and reviewed and edited the manuscript. RS and TD independently performed title and abstract screening and full-text review, independently performed a pilot data extraction on a random sample, and subsequently refined the form. RS and a study assistant conducted data extraction independently and met frequently with TD to discuss the process and refine the data extraction form. TD, SU, and TF critically read and commented on the original draft. All authors read and approved the final manuscript, which was completed in December 2022.
