# Supplementary material for: Identifying patients with psychosocial problems in general practice: A scoping review
Source: Front Med (Lausanne). 2023 Feb 8;9:1010001. doi: 10.3389/fmed.2022.1010001 (PMC9945547; doi:10.3389/fmed.2022.1010001)
Supplement: Supplementary file 1 [file Table_1.docx]

Supplementary Material

**Table 1**. Search strategy for Medline (Ovid)

| 1 | ((psychosocial or social or mental or psychological or emotional) adj3 (problem* or strain* or stress* or distress* or burden* or issue* or pressure* or suffer* or struggle* or difficult*)).ti,ab. |
| --- | --- |
| 2 | ((poverty or financial or food or water or housing or neighbo*rhood or work or unemployment or education or legal) adj problem*).ti,ab. |
| 3 | (((social adj cultural) or (social adj welfare) or (health adj care adj system) or (compliance) or (being adj ill) or (partner* adj behavio*r) or (partner* adj illness*) or ((loss or death) adj3 partner*) or ((loss or death) adj3 child*) or ((assault or harmful) adj event)) adj (problem*)).ti,ab. |
| 4 | ((relationship* adj problem* adj2 partner*) or (relationship* adj problem* adj2 child*) or (illness adj problem* adj2 child*) or (relationship* adj problem* adj (parent* or family)) or (behavio* adj problem* adj (parent* or family)) or (illness adj problem* adj (parent* or family)) or ((loss or death) adj (parent* or family) adj member*) or (relationship* adj problem* adj friend*) or (fear adj2 social adj problem*) or (limited adj (function or disability))).ti. |
| 5 | exp life change events/ |
| 6 | (complex adj3 health adj3 social).ti,ab. |
| 7 | exp psychosocial deprivation/ |
| 8 | or/1-7 |
| 9 | exp patient health questionnaire/ |
| 10 | (identifi* or detect* or screen* or recogni* or discover*).ti. |
| 11 | ((tool* or instrument* or questionnaire* or interview*) or (medical adj interview*) or (history adj taking)).ti,ab. |
| 12 | exp mass screening/ |
| 13 | biopsychosocial*.ti,ab. |
| 14 | or/9-13 |
| 15 | exp family practice/ |
| 16 | Physicians, Family/ |
| 17 | exp general practice/ |
| 18 | exp family health/ |
| 19 | (((general or family) adj (practi* or physician* or doctor or clinician or medicine*)) or (nurse* adj practition*)).ti,ab. |
| 20 | or/14-19 |
| 21 | 8 and 14 and 20 |
