# Supplementary material for: Identifying patients with psychosocial problems in general practice: A scoping review
Source: Front Med (Lausanne). 2023 Feb 8;9:1010001. doi: 10.3389/fmed.2022.1010001 (PMC9945547; doi:10.3389/fmed.2022.1010001)
Supplement: Supplementary file 2 [file Table_2.pdf]

Supplementary Material

Table 2. Included studies

Study	Title
Corser 1978	Emotional disturbance in newly registered general practice patients
Hilliard 1986	Validity of two psychological screening measures in family practice: Personal Inventory and Family APGAR
McDowell 1987	Screening for psychosocial problems among primary care patients: a pilot study
Corney 1988	Development and use of a short self-rating instrument to screen for psychosocial disorder
Hase 1988	Screening for psychosocial problems in primary care
Bingham 1990	Establishing a quality improvement process for identification of psychosocial problems in a primary care practice
McEwan 1990	Screening elderly people in primary care: a randomised controlled trial
Shiber 1990	Detection of emotional problems in the primary care clinic
Verhaak 1990	The importance of the GHQ in general practice
Forde 1992	Antenatal care in general practice. A questionnaire as a clinical tool for collection of information on psychosocial conditions
Verhaak 1992	Psychosocial problems in primary care: some results from the Dutch national study of morbidity and interventions in general practice.
Gunther 1993	A continuous quality improvement cycle for teaching the identification of psychosocial problems to general internal-medicine residents
Hansson 1994	Screening for psychiatric illness in primary care. A cross-sectional study in a Swedish health district
Stefansson 1994	Identified and unidentified mental illness in primary health care—social characteristics, medical measures and total care utilization during one year
Al-Shammari 1994	Screening for psychosocial problems among primary care patients in Riyadh, Saudi Arabia
Hopton 1995	Measuring psychological well-being. The adapted General Well-Being Index in a primary care setting: a test of validity
Cook 1996	Screening for social and environmental problems in a VA primary care setting
Junius 1996	A prevention program for health problems in the elderly. Ambulatory Geriatric Screening (AGES) for use in general practice
De la Revilla 1997	A method of detection psychosocial problems at the family physician's office
Gulbrandsen 1997	General practitioners' knowledge of their patients' psychosocial problems: multipractice questionnaire survey
Odell 1997	Determinants of general practitioner recognition of psychological problems in a multi-ethnic inner-city health district
Root 1998	Modelling the process of encounter between general practitioner and patient as a method for the detection of psychosocial problems
Smith 1998	The role of the general health questionnaire in general practice consultations

Van der Pasch 1998	Communication in general practice: recognition and treatment of mental illness
Sandholzer 1999	Early diagnosis and early treatment of cognitive disorders: a study of geriatric screening of an unselected patient population in general practice
Raine 2000	Patient determinants of mental health interventions in primary care
Wasson 2000	Routine, single-item screening to identify abusive relationships in women
Deliège 2001	A classification system of social problems: concepts and influence on GPs' registration of problems
Richardson 2002	Identifying domestic violence: cross sectional study in primary care
Watts 2002	Mental health in older adult recipients of primary care services: is depression the key issue? Identification, treatment and the general practitioner
Carr-Gregg 2003	Risk-taking behaviour of young women in Australia: screening for health-risk behaviours
De la Revilla Ahumada 2004	Use of the Goldberg General Health Questionnaire (GHQ-28) to detect psychosocial problems in the family physician's office
Goodyear-Smith 2004	Lifestyle screening: development of an acceptable multi-item general practice tool
Kapur 2004	Psychosocial and illness related predictors of consultation rates in primary care—a cohort study
Saltini 2004	Decisional strategies for the attribution of emotional distress in primary care.
Brotons 2005	Assessment of the effectiveness of an instrument to identify health and social problems in an elderly population from a primary health care center
Goodyear-Smith 2005	Ethnic differences in mental health and lifestyle issues: results from multi-item general practice screening
Kendrick 2005	A trial of problem-solving by community mental health nurses for anxiety, depression and life difficulties among general practice patients. The CPN-GP study
Rabinowitz 2005	Primary care physicians' detection of psychological distress among elderly patients
King 2006	The health of people classified as lesbian, gay and bisexual attending family practitioners in London: a controlled study
Knishkowy 2006	Preventive adolescent health care in family practice: a program summary
Martinez 2006	Factors that influence the detection of psychological problems in adolescents attending general practices
Mirza 2006	Eliciting explanatory models of common mental disorders using the Short Explanatory Model Interview (SEMI) Urdu adaptation—a pilot study
Terluin 2006	The Four-Dimensional Symptom Questionnaire (4DSQ): a validation study of a multidimensional self-report questionnaire to assess distress, depression, anxiety and somatization
Schreuders 2007	Primary care patients with mental health problems: outcome of a randomised clinical trial
Goodyear-Smith 2008	Case finding of lifestyle and mental health disorders in primary care: validation of the 'CHAT' tool
Piccoliori 2008	Geriatric assessment in general practice using a screening instrument: is it worth the effort? Results of a South Tyrol study

Goodyear-Smith 2009	Asking for help is helpful: validation of a brief lifestyle and mood assessment tool in primary health care
Haller 2009	The identification of young peoples’ emotional distress: a study in primary care
MacMillan 2009	Screening for intimate partner violence in health care settings: a randomised trial
Vidotto 2010	Family Strain Questionnaire - Short Form for nurses and general practitioners
Goncalves 2011	Determinants of common mental disorders detection by general practitioners in primary health care in Brazil
Hassink-Franke 2011	Effectiveness of problem-solving treatment by general practice registrars for patients with emotional symptoms
Freund 2012	The effect of preventive consultations on young adults with psychosocial problems: a randomised trial
Hegarty 2012	Identifying intimate partner violence when screening for health and lifestyle issues among women attending general practice
Frese 2013	Feasibility, understandability, and usefulness of the STEP self-rating questionnaire: results of a cross-sectional study
Goodyear-Smith 2013	eCHAT for lifestyle and mental health screening in primary care
Hegarty 2013	Screening and counselling in the primary care setting for women who have experienced intimate partner violence (WEAVE): a cluster randomised controlled trial
Blom 2014	Effectiveness and cost-effectiveness of a proactive, goal-oriented, integrated care model in general practice for older people. A cluster randomised controlled trial: Integrated Systematic Care for older People-the ISCOPE study
Sanci 2015	Responding to young people’s health risks in primary care: a cluster randomised trial of training clinicians in screening and Motivational Interviewing
Webb 2015	Designing a health screening tool to help young people communicate with their general practitioner
Tak 2016	Development and preliminary validation of an Observation List for detecting mental disorders and social Problems in the elderly in primary and home care (OLP)
Ambresin 2017	Training general practitioners to detect probable mental disorders in young people during health risk screening
Geyti 2018	Initiation and cessation of mental healthcare after mental health screening in primary care: a prospective cohort study
Geyti 2020	Factors associated with non-initiation of mental healthcare after detection of poor mental health at a scheduled health check: a cohort study
Klein 2020	Screening and counselling adolescents and young adults: a framework for comprehensive care
