# Supplementary material for: Identifying patients with psychosocial problems in general practice: A scoping review
Source: Front Med (Lausanne). 2023 Feb 8;9:1010001. doi: 10.3389/fmed.2022.1010001 (PMC9945547; doi:10.3389/fmed.2022.1010001)
Supplement: Supplementary file 3 [file Table_3.docx]

Supplementary Material

**Table 3**. Data extraction form

| General information | Lead author & year of publication |
| --- | --- |
|  | Title |
| Characteristics of included evidence sources | Type of evidence source (e.g., study, instrument description) |
|  | Research design / study type |
|  | Aim(s) described in publication |
|  | Relevant part for our review |
|  | Setting |
|  | Country |
| Population | Population described (e.g., patients, citizens, residents, physicians) |
|  | Specific characteristics (e.g., geriatric patients, pregnant women, young people) |
|  | Inclusion criteria |
|  | Exclusion criteria |
|  | Total number of analysed research participants |
|  | Age (years) |
|  | Gender |
| Concept: Description of identification instrument(s) | Term used to refer to ‘psychosocial problems’ (e.g., mental health problems, psychological distress) |
|  | Name of instrument(s) |
|  | Aim of instrument(s) |
|  | Description |
|  | Type of instrument (e.g., questionnaire, interview) |
|  | Type of reporting format (e.g., self-reported, physician’s assessment, clinical examination) |
|  | Instrument administered by (e.g., physician, nurse, other professional, patient) |
|  | Components examined (if reported) (e.g., social factors, risk factors, protective factors) |
|  | Regularity / conduction of screening (if reported) (e.g., initial screening, routine screening of all patients, only those at risk) |
| Findings | Conclusion stated by authors |
|  | Key points that relate to our research question |
