# Supplementary material for: Identifying patients with psychosocial problems in general practice: A scoping review
Source: Front Med (Lausanne). 2023 Feb 8;9:1010001. doi: 10.3389/fmed.2022.1010001 (PMC9945547; doi:10.3389/fmed.2022.1010001)
Supplement: Supplementary file 4 [file Table_4.pdf]

Supplementary Material

Table 4. General study characteristics

Study	Country	Study type	Age group	Population	Sample size	Female sex, %	Study aim(s)	Name of instrument(s)	Author's conclusions
Corser 1978	England, UK	Cross-sectional study	Adults	Pat	119	52.9	Look at the emotional state of recently registered newcomers to a new town, ascertain whether mental state was related to the demographic composition of the registered group, compare high and low GHQ scorers on their use of their GP	General Health Questionnaire (GHQ-60)	- GHQ has acceptable validity, but one cannot determine how long its classification of an individual will hold good<br>- GHQ and similar instruments should not be used to give a permanent index of psychological disability<br>- As a survey instrument the GHQ is useful but whether what it measures is best considered as psychiatric illness or as part of the normal range of emotional responses to life events remains to be clarified

Study	Country	Study type	Age group	Population	Sample size	Female sex, %	Study aim(s)	Name of instrument(s)	Author's conclusions
Hilliard 1986	USA	Prospective study	Adults	Pat	150	73	Determine the level of accuracy with which APGAR identify patients with psychological distress	Personal Inventory, Family APGAR	- Personal Inventory and Family APGAR enhance physicians' ability to recognise emotional distress without substantially extending consultation time and can be incorporated nicely into routine health care visits<br>- Personal Inventory performed better than Family APGAR; results suggest tandem use of instruments
McDowell 1987	Canada	Pilot study	Adults	Pat, Phy, Res	Pat: 724<br>Phy: 9<br>Res: 35	Pat: 64	Test feasibility and acceptability of having patients complete a brief health index questionnaire designed to help them describe their underlying concerns	Brief health index questionnaire	- Patients: questions served well to initiate discussions, high acceptability<br>- Physicians: 41 % of the questionnaires were rated as helpful to identify patients' concerns more rapidly, reveal patients' real agenda for consultation, confirm psychosocial conditions, and to

Study	Country	Study type	Age group	Population	Sample size	Female sex, %	Study aim(s)	Name of instrument(s)	Author's conclusions
									'break the ice' in pertinent but sensitive areas; wide variation in acceptability
Corney 1988	England, UK	Survey	Adults	Pat, Phy	Pat: 100 Phy: 3	Pat: 79	Describe the use of an instrument combining a shortened version of SPQ with GHQ-12 designed to identify psychosocial disorder	No specific name	- Instrument simple to administer and readily acceptable to patients; could be completed within a short period of time; may be particularly useful with newly registered patients, those whom the doctor knows little about, or more reticent patients who find it difficult or embarrassing to voice their problems directly
Hase & Luger 1988	USA	Literature / Narrative review	Adults	Pat	N/A	N/A	Present the use of the screening instrument MHI	Multifactor Health Inventory (MHI)	NR
Bingham 1990	USA	Intervention study	Adults	Pat	204	62	Present a quality improvement process that will significantly increase the rate of identification of psychosocial problems through	Patient-Reported Outcomes Measurement Information System - Adult Medical History Questionnaire for Problem Oriented	- PROMIS database can be used to signal presence of psychosocial problems<br>- Results indicate that regular use of a psychosocial case-

Study	Country	Study type	Age group	Population	Sample size	Female sex, %	Study aim(s)	Name of instrument(s)	Author's conclusions
							routine use of case-finding instruments	Practices (PROMIS III)	finding instrument, with standards and procedures for utilisation that are a routine part of each comprehensive care visit, will effectively identify patients who have psychosocial problems
McEwan 1990	England, UK	Randomised controlled trial	Elderly people	Pat	229	NR	Test the effectiveness of a screening programme carried out by nurses for elderly people	Functional and problem evaluation interview, McMaster health index, Nottingham health profile	- Annual comprehensive screening (home visits, all kinds of problems) may create a high workload for practice team members with little effect on elderly peoples' medical and functional problems<br>- Screening enhances elderly patients' morale<br>- No specific conclusions regarding social/psychosocial problems
Shiber 1990	Israel	Interview study	Adults	Pat, Phy	Pat: 776 Phy: 20	Pat: 56	Measure the prevalence of emotional	General Health Questionnaire (GHQ-28)	- At least half the patients with high levels of emotional

Study	Country	Study type	Age group	Population	Sample size	Female sex, %	Study aim(s)	Name of instrument(s)	Author's conclusions
						Phy: 70	problems in patients and the extent to which physicians detect them		problems were not identified by physicians<br>- GHQ may be 'the' tool for establishing 'caseness' in terms of a screening device, intended to identify patients suffering from emotional problems who warrant further intensive professional investigation
Verhaak 1990	Netherlands	Survey	Adults	Pat	Pat: national study: 8747, week sample: 476	NR	Explore to what degree patients who present with mental problems in general practice, or those diagnosed by the GP as having psychologically induced complaints are detected by the GHQ	General Health Questionnaire (GHQ-30)	- In the present primary care situation, where a large group of undetected mental disorders exists, it seems only attainable for a GP to wait for more conclusive evidence; this might constitute the pertaining use of medical care, by accumulating evidence during subsequent visits, etc.<br>- The combination of intuition and a

Study	Country	Study type	Age group	Population	Sample size	Female sex, %	Study aim(s)	Name of instrument(s)	Author's conclusions
									lifelong relationship with a patient is, perhaps, at least as good as a screening device<br>- It might perhaps in the future be possible to design screening instruments, aimed at reaching a better diagnosis when intervention might be successful, instead of the aim of detecting mental distress without looking at the possibilities of treatment
Forde 1992	Norway	Evaluation of a questionnaire	Adults	Pat*	65	100	Describe and analyse a questionnaire developed for the systematic collection of information on pregnant women's psychosocial conditions	Psychosocial questionnaire	- Systematic approach to pregnant women's psychosocial condition facilitates collection of new and potentially useful information and ensures inclusion of psychosocial conditions as part of standard routines in antenatal care

Study	Country	Study type	Age group	Population	Sample size	Female sex, %	Study aim(s)	Name of instrument(s)	Author's conclusions
Verhaak 1992	Netherlands	Cohort study	Adults	Pat	397	NR	Describe the treatment and natural course of psychological problems in general practice in terms of needs and demands	General Health Questionnaire (GHQ-30), Biographical Problem Inventory	- As results do not indicate that specialists in mental health care are more successful than GPs, it does not seem fair to place blame for this situation on GPs<br>- On the contrary, though general practice should do better still, most energy was spent on those patients who 'objectively' needed it most. The task in specialists' hands is perhaps to keep on trying to provide general practice with better tools for those areas where yet more is to be gained
Gunther & Bingham 1993	USA	Intervention study	Adults	Pat, Res	Pat: 264<br>Res: 34	NR	Study a Continuous Quality Improvement (CQI) cycle intended to enhance residents'	Review-of-systems questionnaire	- Identification and treatment planning of psychosocial problems is continually reinforced and reviewed throughout the CQI

Study	Country	Study type	Age group	Population	Sample size	Female sex, %	Study aim(s)	Name of instrument(s)	Author's conclusions
							identification of psychosocial problems		approach training program<br>- Outcome of the study indicates that the regular use of psychosocial case-finding instruments within a CQI cycle can be an effective tool for educating residents in primary care and helps to enhance detection rates for psychosocial problems
Hansson 1994	Sweden	Cross-sectional study	Adults	Pat, Phy	Pat: 388 GPs: 22	Pat: 51.3	Investigate the frequency of psychiatric illness, analyse characteristics of patients with psychiatric illness with regard to socio-demographic characteristics, utilisation of care, psychosocial and environmental problems	No specific name	- Psychosocial worries and problems are frequently reported by patients, particularly regarding work situation, private economic situation, loneliness, social isolation, and unemployment<br>- Findings point to interaction of psychological impairment, environmental stress, coping skills, and

Study	Country	Study type	Age group	Population	Sample size	Female sex, %	Study aim(s)	Name of instrument(s)	Author's conclusions
									social support of the patient as being of importance to the distinction between psychiatric cases and non-cases<br>- Evaluation of such factors by GPs may be of considerable relevance to the detection of psychiatric illness in primary care
Stefansson & Svensson 1994	Sweden	Cross-sectional study	Adults	Pat	333	65.8	Screen patients with regard to identified and unidentified mental illness and describe social and demographic profiles	Hopkins-Symptom-Checklist (HSCL-25)	- Social problems are present to a high degree in GP patients suffering from mental illness (identified or unidentified)<br>- Problems were chiefly centred around employment, personal finances, and social isolation
Al-Shammari 1994	Saudi Arabia	Cross-sectional study	Adults	Pat, Phy	Pat: 582<br>Phy: NR	Pat: 57.6	Explore the psychosocial problems experienced by patients and compare doctors'	Self-reporting Questionnaire (SRQ-20), Social Problem Questionnaire (SPQ)	- Instrument was found to be simple to administer and readily accepted by patients<br>- Although the instruments used are no substitute for a

Study	Country	Study type	Age group	Population	Sample size	Female sex, %	Study aim(s)	Name of instrument(s)	Author's conclusions
							identification of such problems		doctor's personal assessment, they certainly uncover problems that require further, delicate probing by the doctor<br>- Particularly useful with reticent patients who find it difficult and/or embarrassing to discuss personal problems directly, especially problems involving intimate relationships<br>- Use of a questionnaire serves to enhance patients' sense of satisfaction that their health needs are receiving adequate attention<br>- Problems such as relationship difficulties, especially in marriage, were more likely to be identified by the questionnaire than by doctors

Study	Country	Study type	Age group	Population	Sample size	Female sex, %	Study aim(s)	Name of instrument(s)	Author's conclusions
Hopton 1995	Scotland, UK	Survey	Adults	Pat	249	56.7	Examine the validity of the AGWBI	Adapted General Well-Being Index (AGWBI)	- AGWBI has potential to measure positive psychological well-being, improvements in mental health, and low levels of psychological distress
Cook 1996	USA	Survey	Adults	Pat**	132	22.7	Obtain information on social and environmental problems of patients at a Veterans Affairs (VA) medical centre	Social Needs Checklist (SNC)	- Nearly all PC patients reported having one or more social problems, the most prevalent being financial difficulties, personal stress, family problems, and legal concerns<br>- When given the opportunity, nearly one in three patients in the PC clinic asked to see a social worker
Junius 1996	Germany	Presentation, description	Elderly people	Pat	463	71	Present the development and main results of the screening project and share the standardised examination instrument with	Ambulatory Geriatric Screening (AGES)	- Outpatient geriatric screening helps to better identify the assistance needs of elderly patients in PC<br>- Study has shown that a high percentage of previously undetected problems

Study	Country	Study type	Age group	Population	Sample size	Female sex, %	Study aim(s)	Name of instrument(s)	Author's conclusions
							interested physicians		can be detected at an early stage by means of a standardised examination. GPs can then check the results at a glance and adjust discussion and procedures accordingly<br>- Physicians were least aware of the psychosocial problems of their elderly patients
De la Revilla 1997	Spain	Crossover study	Adults	Pat	202	68.3	Find the value of SVE for detecting patients with psychosocial problems and how these affect family function	General Health Questionnaire (GHQ-28), Stressful Vital Events (SVE), Family APGAR	- Exploration of stressful life events is a good way to detect psychosocial problems<br>- Authors suggest the convenience of routinely using the social readjustment scale, together with social support questionnaires to detect clinical problems in the consultation room that have their origin in social stress

Study	Country	Study type	Age group	Population	Sample size	Female sex, %	Study aim(s)	Name of instrument(s)	Author's conclusions
Gulbrandsen 1997	Norway	Survey	Adults	Pat, Phy	Pat: 1,217 Phy: 89	Pat: 64	Evaluate GPs' awareness of a range of problems among patients and explore whether doctors' recognition depends on previous knowledge about the patient, the type of problem, or characteristics of patient and doctor	No specific name	- At least one third of patients in general practice have psychosocial problems that they perceive as influencing their present health<br>- GPs recognise a fifth to a half of these problems, depending on its type, their previous general knowledge, and the sociodemographic characteristics of the patient<br>- Variation in the patients' wishes and abilities to communicate, the need for confidence in the doctor-patient relationship before revealing intimate problems, and a tendency for doctors to be entrapped by their expectations may be some reasons for these findings

Study	Country	Study type	Age group	Population	Sample size	Female sex, %	Study aim(s)	Name of instrument(s)	Author’s conclusions
Odell 1997	England, UK	Cross-sectional study	Adults	Pat, Phy	Pat, Phy: 833	Pat: 66 Phy: 21	Identify those factors that contribute to GPs’ detection of psychological complaints and examine how these factors influence their correct identification	GHQ-30	- It seems likely that awareness of social problems facilitates recognition of psychological problems and that social problems may be a criterion used by GPs to make a psychiatric diagnosis<br>- Findings suggest that there is a particular need to consider different cultural explanations of mental distress
Root & Maoz 1998	Netherlands	Presentation, description	Adults	Pat, Phy	N/A	N/A	Explain the consultation model as one that structures the process of consultation	Structured Consultation Model (SOAP & BATHE)	- It can be very helpful for a GP to pay attention to modelling the consultation process in an open but structured way<br>- The process of consultation and handling it in a systematic and structured way is much more important to the GP than thinking and working

Study	Country	Study type	Age group	Population	Sample size	Female sex, %	Study aim(s)	Name of instrument(s)	Author's conclusions
									in a conceptual framework based on psychosomatic or psychosocial theories
Smith 1998	Scotland, UK	Randomised controlled trial	Adults	Pat, Phy	Pat: 1382 Phy: 8	Pat: 67.1 Phy: 50	Determine the practical application of GHQ during a routine consultation and assess its effect on recognition of emotional distress	General Health Questionnaire (GHQ-30), No specific name ('Doctors questionnaire')	- GHQ could be an acceptable addition to the consultation because its use does facilitate the identification of patients who are emotionally distressed<br>- The large proportion of questionnaires that were ignored raises concerns both for doctor and for patient acceptability<br>- GHQ is well validated for use in a GP setting. It takes only a few minutes for the patient to complete and the score can be easily totalled by the doctor during the consultation<br>- In this study, as in previous studies based in general practice,

Study	Country	Study type	Age group	Population	Sample size	Female sex, %	Study aim(s)	Name of instrument(s)	Author's conclusions
									GHQ was associated with a significant increase in the detection of emotional distress
Van der Pasch & Verhaak 1998	Netherlands	Secondary analysis	Adults	Pat, Phy	Pat: 808 Phy: 15	NR	Report on a study exploring GPs' ability to recognise mental illness	General Health Questionnaire (GHQ-12)	- An 'accurate' doctor detects relatively many mentally disturbed patients, but should also recognise emotional disturbance in mentally healthy patients<br>- Attention to emotional disturbances that are not psychiatric illnesses in the narrow sense is equally important
Sandholzer 1999	Germany	Cross-sectional study	Elderly people	Pat, Phy	Pat: 446 Pract: 67	Pat: 71.5	Investigate the feasibility of early assessment of preventable disabilities: a geriatric preventive screening examination with various indicators of	Ambulatory Geriatric Screening (AGES)	- Through screening, many social findings with disease value and problem severity were discovered for the first time<br>- The highest detection rate is found in the area of psychological, especially cognitive,

Study	Country	Study type	Age group	Population	Sample size	Female sex, %	Study aim(s)	Name of instrument(s)	Author's conclusions
							physical, emotional, social functions, and laboratory exams		impairments, followed by social or functional limitations
Raine 2000	England, UK	Cross-sectional study	Adults	Pat	802	62.1	Identify and compare the influence of non-clinical patient factors on GPs' acknowledgement of mental problems and on their provision of mental health care	General Health Questionnaire-28 (GHQ-28), Short Form-36 (SF-36)	- Mental problems are common in PC and their acknowledgement is a necessary but not a sufficient condition for intervention<br>- Findings suggest that the likelihood of intervention is not a direct consequence of a GP's acknowledgement of the presence of mental problems
Wasson 2000	USA	Survey	Adults	Pat	1,526	100	Reemphasise why specific inquiry about an abusive relationship is important and how it might be accomplished in a busy office setting	Patient Health Survey	- Domestic abuse is a prevalent and important problem of women and simple measures can detect domestic abuse in community practice<br>- It is necessary to think of abuse when some functional and symptomatic issues are present, but that

Study	Country	Study type	Age group	Population	Sample size	Female sex, %	Study aim(s)	Name of instrument(s)	Author's conclusions
									alone is not sufficient; direct inquiry will be more effective
Deliège 2001	Belgium	Mixed methods: Group discussion, Survey	Adults	Phy	8	NR	Proposing a conceptual framework for psychosocial problems	Coding System for Primary Health Care (CPHC)	- Study resulted in a revised CPHC coding-list introducing extra concepts for classifying problems. Psychological problems within most sections: emotions-feelings, behaviour, delays, and disabilities. Social problems (type of problem) within most domains: loss, lack, defect, discord, adaptation to change or to usual requirements<br>- Training GPs to use such a coding system drastically increases the number of psycho-social problems, but only during the prospective phases; in the long run older habits prevail again

Study	Country	Study type	Age group	Population	Sample size	Female sex, %	Study aim(s)	Name of instrument(s)	Author's conclusions
Richardson 2002	England, UK	Cross-sectional study	Adults	Pat	1,207	100	Measure the prevalence of domestic violence among women, test the association between experience of domestic violence and demographic factors, measure the proportion of women experiencing domestic violence that is not detected, and explore women's attitudes to being questioned about domestic violence by GPs or practice nurses	No specific name	- With the high prevalence of domestic violence, health professionals should maintain a high level of awareness of the possibility, especially for pregnant women, but the case for screening is not yet convincing<br>- It seems to be helpful to use the presented questionnaire<br>- It is necessary to screen for domestic violence, because over a third of women attending general practices had experienced physical violence from a male partner
Watts 2002	England, UK	Survey	Elderly people	Pat, Phy, PN	Pat: 268<br>Phy: NR<br>PN: NR	Pat: 61.7	Identify the nature and extent of mental health problems in older people	General Health Questionnaire (GHQ-28), Hospital Anxiety and Depression Scale (HADS)	- Study supports research suggesting that older adults may suffer a range of mental health problems, not

Study	Country	Study type	Age group	Population	Sample size	Female sex, %	Study aim(s)	Name of instrument(s)	Author's conclusions
									restricted to depression and organic problems, and may be reluctant to mention psychological distress to GP staff<br>- Study also raises concerns about validity of simple screening at a single point in time as a method of identifying individuals in need of mental health treatment, and emphasises the complexity of diagnosis in the context of a brief surgery consultation with a GP or practice nurse
Carr-Gregg 2003	Australia	Literature / Narrative review	Adolescents	Pat	N/A	N/A	Review the prevalence of a range of health-risk behaviours, discuss challenges faced by GPs, and present a	HEADSS (Home, education, activities, drugs, sexuality, suicide/depression, safety)	NR

Study	Country	Study type	Age group	Population	Sample size	Female sex, %	Study aim(s)	Name of instrument(s)	Author's conclusions
							psychosocial screening device		
De la Revilla Ahumada 2004	Spain	Cross-sectional study	Adults	Pat	314	69.4	Analyse the usefulness of GHQ-28 in identifying psychosocial problems and determine how the questionnaire scores are related to stressful life events	General Health Questionnaire (GHQ-28)	- GHQ-28 is a potentially useful instrument to detect psychosocial problems, and can aid in the subsequent identification and qualitative evaluation of patients<br>- GHQ-28 is positively related to recent stressful life events and a series of variables that are associated with the appearance of psychosocial processes
Goodyear-Smith 2004	New Zealand	Cross-sectional study	Adults	Pat, Phy, PN	Pat: 2,543<br>Phy: 31<br>PN: 20	Pat: 66.67	Develop a short screening tool for lifestyle and mental-health risk factors, determine acceptability and feasibility	Lifestyle assessment screening tool	- Screening tool was very acceptable to patients in both urban and rural settings and was not considered overly burdensome by practitioners<br>- Initial fears that doctors would be inundated with requests for help with

Study	Country	Study type	Age group	Population	Sample size	Female sex, %	Study aim(s)	Name of instrument(s)	Author's conclusions
									newly identified lifestyle problems, in addition to the scheduled consultation, were not realised
Kapur 2004	England, UK	Prospective study	Adults	Pat	738	59.4	Identify the psychosocial- and illness-related factors that independently predicted primary care consultation over a 5-year period	General Health Questionnaire (GHQ-12)	- Questionnaires seem to be useful to assess outcomes after a period of one or three years. A large part of the instruments were well validated and established in many countries<br>- Terms such as 'frequent attenders' may be less helpful than recognising a number of dimensions that operate across the whole spectrum of consultation frequency
Saltini 2004	Italy	Cross-sectional study	Adults	Pat, Phy	Pat: 444<br>Phy: 6	Pat: 68	Identify those clinical and psychosocial data on patients that increase the likelihood of GPs' attribution	General Health Questionnaire (GHQ-12), Social Problem Questionnaire (SPQ), Stressful Life Events	- The strategy to improve the detection of current emotional distress suggests giving foremost and systematic attention to social problems and

Study	Country	Study type	Age group	Population	Sample size	Female sex, %	Study aim(s)	Name of instrument(s)	Author’s conclusions
							of emotional distress and those that predict patients’ emotional distress as defined by the GHQ-12		recent life events of loss as important clues for the possible presence of emotional problems and to be aware of critical patient data, in particular psychiatric history, psychopharmacological treatment and chronic illness, which increase the probability of attribution errors<br>- Since psychosocial issues are less likely to emerge or to be considered using the traditional biomedical approach, the mastery of patient-centred communication techniques becomes a necessary condition for the correct identification and handling of emotional distress in general practice patients

Study	Country	Study type	Age group	Population	Sample size	Female sex, %	Study aim(s)	Name of instrument(s)	Author's conclusions
Brotons 2005	Spain	Cross-sectional study	Elderly people	Pat	1,299	63.1	Assess the response rate to a multi-dimensional, self-administered questionnaire and establish the prevalences of problems in the following dimensions: socio-economic, cognitive, morbidity, polypharmacy, physical activity, falls, daily living activities	Brief Multidimensional Assessment Questionnaire	- Comprehensive geriatric assessment using the postal method is effective and allows the identification and quantification of multiple socio-health problems in the elderly<br>- It is feasible to identify the most fragile; finding supports their regular implementation in order to carry out preventive and curative programmes in primary care and social care<br>- Use of this instrument makes it possible to make concrete proposals on the health resources needed in relation to a given intervention
Goodyear-Smith 2005	New Zealand	Cross-sectional study	Adults	Pat, Phy	Pat: 1,000<br>Phy: 20	Pat: 66.67	Determine ethnic differences in response, acceptance, and	Multi-item Screening Tool (MIST)	- Screening tool was very well accepted by patients, with a minimal objection

Study	Country	Study type	Age group	Population	Sample size	Female sex, %	Study aim(s)	Name of instrument(s)	Author's conclusions
							desire to address problems identified by the MIST		rate to any of the questions. Greatest objection was to the question on recreational drug use<br>- No ethnic differences in the acceptance of the tool
Kendrick 2005	England, UK	Randomised controlled trial	Adults	Pat, Phy, CMHN	Pat: 247 Phy: 241 CMNH: 53	Pat: 70.4	Compare the effectiveness of community mental health nurse (CMHN) problem-solving and generic CMHN care, against usual GP care in reducing symptoms, alleviating problems, and improving social functioning and quality of life	General Health Questionnaire (GHQ-12), Hospital Anxiety and Depression Scale (HADS)*, Social Adjustment Scale (SAS)*, Problem Appraisal Scale (PAS)*	NR
Rabinowitz 2005	Israel	Survey	Elderly people	Pat, Phy	Pat: 1,007 Phy: 67	Pat: 58.6 Phy: 55.2	Compare rates of psychological distress and physician detection among elderly and non-elderly patients,	General Health Questionnaire (GHQ-28)	- Physicians correctly detect about one third of the GHQ distressed cases; no evidence that physicians detect distress less accurately among

Study	Country	Study type	Age group	Population	Sample size	Female sex, %	Study aim(s)	Name of instrument(s)	Author’s conclusions
							examine variables associated with distress and physician detection		elderly than in younger patients<br>- In this study the GHQ was used to encourage the detection rate of psychological distress; instrument is well-established and seems to be very useful to improve the detection rate of such cases
King 2006	England, UK	Cross-sectional study	Adults	Pat	1.479	70.7	Compare health measures of family practice attendees classified as lesbian, gay, and bisexual	General Health Questionnaire (GHQ-12), Short Form Health Survey 12 (SF-12)	- Increased awareness of the sexuality of people seen in primary care can provide opportunities for health promotion<br>- Both validated instruments gave a good usable result
Knishkowy 2006	Israel	Focus groups, individual discussions	Adolescents	Pat	184	48.9	Give an overview of a family practice-based adolescent preventive health programme and report on compliance,	Community-Oriented Primary Care (COPC) programme	- Psychosocial and behavioural problems such as lack of seatbelt use, unhealthy dieting, smoking and alcohol use, were frequent issues requiring intervention

Study	Country	Study type	Age group	Population	Sample size	Female sex, %	Study aim(s)	Name of instrument(s)	Author's conclusions
							feasibility, and health issues		- Assessing and counselling regarding family problems and depression were also quite common, and the focus of many visits<br>- Using a concerns checklist was an invaluable tool that turned the visits into 'patient-oriented' encounters; it shows the teenager that the health team is ready to discuss a wide range of issues and allows them to make their personal concerns the focus of the visit
Martinez 2006	England, UK	Prospective study	Adults	Pat, Phy	Pat: 98; Par: 76 Phy: 13	Pat: 61.2 Phy: 46.2	Identify factors that influence the detection of psychological difficulties	Strengths and Difficulties Questionnaire (SDQ)	- SDQ is a screening instrument rather than a diagnostic tool<br>- Open discussion of psychological issues in GPs' consultations with adolescents is relatively uncommon, yet this is the most important factor

Study	Country	Study type	Age group	Population	Sample size	Female sex, %	Study aim(s)	Name of instrument(s)	Author's conclusions
									influencing detection of psychological problems in adolescents<br>- It appears that GPs and adolescents are reluctant to explore psychological issues in the consultation, even when they both suspect that these are important
Mirza 2006	Pakistan	Pilot study	Adults	Pat	15	33.33	Field test the SEMI-Urdu adaptation to generate preliminary data on explanatory models of mental distress, describe the presenting symptoms and their explanation from the patients' perspective	General Health Questionnaire (GHQ-12)	- Brief version of SEMI-Urdu adaptation is relatively simple to administer, helps to quantify patient's view of the condition and to develop a shared language between patient and provider<br>- All GHQ-12 cases presented for non-specific reasons and regarded their problem as so intense that it limits their functioning at home, work, and in the

Study	Country	Study type	Age group	Population	Sample size	Female sex, %	Study aim(s)	Name of instrument(s)	Author's conclusions
									social arena, and identified their current presenting problems as arising in their social world
Terluin 2006	Netherlands	Secondary analysis	Adults	Pat	7,403	55.1	Evaluate criterion and construct validity of the 4DSQ	Four-Dimensional Symptom Questionnaire (4DSQ)	- The notion that distress is the most general expression of psychological problems of any kind was confirmed; Distress score showed substantial correlations with the scores of various other questionnaires measuring a range of symptoms from distress to depression and from anxiety to somatization; Distress score was associated with psychosocial stressors, and especially with psychosocial problems such as marital and financial problems and excessive occupational demands

Study	Country	Study type	Age group	Population	Sample size	Female sex, %	Study aim(s)	Name of instrument(s)	Author’s conclusions
									- Findings support the assumption that the Distress score is a non-specific indicator of any psychological problem.<br>- Distress score was also shown to be the most important predictor of social dysfunctioning and sick leave<br>- It is plausible that distress plays a role in motivating patients to seek help and to discuss psychological issues with their doctor<br>- One of the practical applications of the 4DSQ in general practice is to increase patients’ awareness of their distress, and to encourage their acknowledgement of psychological problems and their readiness to discuss

Study	Country	Study type	Age group	Population	Sample size	Female sex, %	Study aim(s)	Name of instrument(s)	Author's conclusions
									these problems with their doctor<br>- 4DSQ appears to be a valid self-report questionnaire to measure distress, depression, anxiety, and somatization in primary care
Schreuders 2007	Netherlands	Randomised controlled trial	Adults	Pat, PN	Pat: 130<br>PN: 12	Pat: 70.8	Investigate the effectiveness of problem-solving treatment provided by mental health nurses	General Health Questionnaire (GHQ-12), Hospital Anxiety and Depression Scale (HADS)*, Patient Health Questionnaire (PHQ)*, Short-Form 36 (SF-36)*	NR
Goodyear-Smith 2008	New Zealand	Validation study	Adults	Pat	1,000	71	Assess criterion-based validity of CHAT against a composite gold standard	Case-finding and Help Assessment Tool (CHAT)	- CHAT tool is both valid and acceptable for lifestyle and mental health disorder screening<br>- All items showed good sensitivity, specificity, and likelihood ratios when compared with gold standard instruments,

Study	Country	Study type	Age group	Population	Sample size	Female sex, %	Study aim(s)	Name of instrument(s)	Author's conclusions
									except for exercise and eating disorders<br>- CHAT provides an important tool for routine use in primary health care settings in lifestyle and mental health domains where a strong argument can be made for case finding and subsequent intervention<br>- Some practices are already using it with all new patients, and asking adult patients to complete it if it has been more than 2 years since their last visit
Piccoliori 2008	Italy	Cross-sectional study	Elderly people	Pat, Phy	Pat: 894 Phy: 45	Pat: 61 Phy: 11.1	Examine whether geriatric assessment could detect problems unknown in general practice, if previously unknown problems were seen as important	Standardised Assessment of Elderly People in primary care (STEP)	- Geriatric assessment as a screening-instrument is able to identify a number of important illnesses, diseases and disabilities—even in general practice where patients are

Study	Country	Study type	Age group	Population	Sample size	Female sex, %	Study aim(s)	Name of instrument(s)	Author's conclusions
							by GPs and patients, whether the length of the GP–patient relationship influenced the number of problems identified, and the consequences for the patient in identifying problems		usually quite well known<br>- The longer a patient is known, the fewer new problems can be identified by screening<br>- Patients and doctors differ remarkably in which of the newly identified health problems they see as important and worth the effort to intervene<br>- GPs focussed more on medical issues, while patients focussed more on psychosocial issues<br>- A high percentage of newly identified problems were approached by doctor and patient and were seen as being 'treated' successfully 1 year after the screening
Goodyear-Smith 2009	New Zealand	Secondary analysis	Adults	Pat	755	NR	Assess the additional value of the help question for each	Case-finding and Help Assessment Tool (CHAT)	- Help question increased specificity without compromising

Study	Country	Study type	Age group	Population	Sample size	Female sex, %	Study aim(s)	Name of instrument(s)	Author's conclusions
							of the individual items in terms of estimating diagnostic accuracy		sensitivity and reduced false positives, thereby increasing the positive predictive value. It allowed patients with comorbidities to prioritise issues they wished to address, indicate their readiness to change, promote self-determination, and give the clinician an indication of which topics to pursue<br>- Using the help question reduces the consultation time needed to determine which patients require immediate intervention<br>- False-positive CHAT responses may represent patients with subsyndromal conditions<br>- CHAT can be used to identify at-risk patients for whom

Study	Country	Study type	Age group	Population	Sample size	Female sex, %	Study aim(s)	Name of instrument(s)	Author's conclusions
									education, primary prevention, and early intervention can be provided to improve health. As a simple, efficient, and validated tool well-suited to the resource- and time-strapped primary care environment, it allows health care clinicians to rapidly assess the important mental and social needs of their patients
Haller 2009	Australia	Cross-sectional study	Young people	Pat, Phy	Pat: 450 Phy: 106	Pat: 66 Phy: 41	Provide a detailed description of the factors associated with both 'correct' and 'excessive' identification of youth mental disorder	Kessler's Scale of emotional distress (K10)	- GPs typically identified mental disorder in those who perceived they had a mental illness and who expressed fears in relation to their health problem<br>- Continuity of care and days out of role favoured identification, but also appeared to favour over-identification of those who were

Study	Country	Study type	Age group	Population	Sample size	Female sex, %	Study aim(s)	Name of instrument(s)	Author's conclusions
									unlikely to have a mental disorder
MacMillan 2009	Canada	Randomised controlled trial	Adults	Pat	411	100	Determine the effectiveness of IPV screening and communication of positive results to clinicians	Woman Abuse Screening Tool (WAST), Composite Abuse Scale (CAS)*, World Health Organization Quality of Life (WHOQOL)-Bref instrument*, Short Form Health Survey (SF-12)*	- Results of this study suggest that use of a specific written, self-completed IPV screen with female adult patients presenting to clinical settings within a universal health care system leads to a few modest benefits and is not associated with short-term harms<br>- The use of all screening instruments mentioned seems to be helpful and effective to get an overview of the problem of 'intimate partner violence'; entirety of instruments make it possible to identify women who suffer from domestic violence
Vidotto 2010	Italy	Survey	Adults	Care	40	77.5	Present an instrument for	Family Strain Questionnaire	- Study confirms that the FSQ-SF offers an

Study	Country	Study type	Age group	Population	Sample size	Female sex, %	Study aim(s)	Name of instrument(s)	Author's conclusions
							recognising the psychological problems of caregivers	Short Form (FSQ-SF)	initial measure of caregivers' psychological status, regardless of the patient's disease and allows professionals to monitor it over time with a rapid and effective instrument<br>- Overworked health professionals, especially those who work in patients' homes, need a screening tool that can be administered and interpreted quickly while still constituting a useful tool<br>- FSQ-SF may give practitioners a quick but reliable indicator as to whether caregivers should be referred to services providing expert psychological help
Goncalves 2011	Brazil	Cross-sectional study	Adults	Pat	714	74.1	Evaluate the common mental disorder detection rates by	General Health Questionnaire (GHQ-12)	- GHQ-12 gives case probability instead of a diagnosis which could be obtained

Study	Country	Study type	Age group	Population	Sample size	Female sex, %	Study aim(s)	Name of instrument(s)	Author's conclusions
							physicians and factors that affect detection		from standardised interviews such as the Composite International Diagnostic Interview or semi-structured instruments such as the Clinical Interview Schedule<br>- GHQ has good sensitivity for primary health care cases, being suitable for identifying common mental disorders
Hassink-Franke 2011	Netherlands	Controlled clinical trial	Adults	Pat, Phy	Pat: 202 Phy: 81	Pat 70.3 Phy: 70.4	Study the effectiveness of problem-solving treatment delivered by trained GP registrars for patients with emotional symptoms	General Health Questionnaire (GHQ-12), Patient Health Questionnaire (PHQ)*, Hospital Anxiety and Depression Scale (HADS)*, Short Form-36 (SF-36)*, 5-dimension EuroQol (EQ-5D)*	- All utilised questionnaires are well-known in research and often used; it can be assumed that they reliably measure the corresponding construct
Freund 2012,	Denmark	Randomised controlled trial	Young people	Pat	364	76	Evaluate the effect of preventive health	No specific name ('Pre-trial screening	- Questionnaires were mainly used to prime the patient before the

Study	Country	Study type	Age group	Population	Sample size	Female sex, %	Study aim(s)	Name of instrument(s)	Author's conclusions
Freund & Lous 2012							consultations on health-related quality of life and lifestyle among 20 to 44-year-olds with multiple psychosocial and lifestyle problems	questionnaire'), No specific name ('Baseline questionnaire')*, Short Form-12 (SF-12)*	consultation and only for some validated questions to compare their status before and after the intervention<br>- Completing the questionnaire was supposed to facilitate insight into the relationship between social life, health, lifestyle, own reaction to stressors and resources, barriers for gaining control and changing behaviour<br>- This insight made it easier for the GPs to offer patient-centred counselling and ask questions that offer a holistic picture of the participant
Hegarty 2012	Australia	Cross-sectional study	Adults	Pat, Phy	Pat: 9,742 Phy: 55	Pat: 100 Phy: 60	Explore the association between fear of a partner or ex-partner and important health and lifestyle issues, compare	No specific name	- Study has shown that health professionals working in PC need to be alert to the fact that IPV is associated with several risk factors for

Study	Country	Study type	Age group	Population	Sample size	Female sex, %	Study aim(s)	Name of instrument(s)	Author's conclusions
							women's comfort to discuss health and lifestyle issues and openness to seek help from GPs and practice nurses		morbidity and mortality<br>- Therefore, clinicians should be aware that if they are prompted to ask about prevention and risk factors, then also asking about fear of a partner is appropriate<br>- Survey is useful in identifying which women are affected by intimate partner violence and it is thus useful in increasing the rate of detection of abuse and expanding the possibilities for identification and early intervention
Frese 2013	Germany	Cross-sectional study	Elderly people	Pat, Phy	Pat: 987<br>Phy: 28	Pat: 59.9	Evaluate acceptability, feasibility, comprehensibility, usefulness of the self-rated version of STEP by patients and GPs	Standardised Assessment of Elderly People in primary care (STEP)	- Self-rating STEP questionnaire is particularly useful with previously unknown patients, patients known for only a short time, or patients irregularly encountered

Study	Country	Study type	Age group	Population	Sample size	Female sex, %	Study aim(s)	Name of instrument(s)	Author's conclusions
									- STEP is generally feasible and accepted by a majority of older patients in a routine GP setting<br>- STEP does not only identify problems that may be successfully treated: remarkably, newly identified psychosocial problems were not followed by any consequences
Goodyear-Smith 2013	New Zealand	Mixed methods	Adults	Pat, Phy	Pat: 196<br>Phy: 7<br>PN: 4<br>Other: 5	NR	Assess the feasibility and acceptability of the systematic use of eCHAT	Electronic Case-finding and Help Assessment Tool (eCHAT)	- eCHAT is an acceptable and feasible means of systematic screening of PC patients for unhealthy behaviours and negative mood states; it is easily integrated into the PC electronic health record<br>- eCHAT can be easily incorporated into the family practice environment as most patients, physicians, and other

Study	Country	Study type	Age group	Population	Sample size	Female sex, %	Study aim(s)	Name of instrument(s)	Author's conclusions
									practice staff find it acceptable for use in this setting
Hegarty 2013	Australia	Cluster randomised trial	Adults	Pat, Phy	Pat: 272 Phy: 52	Pat: 100 Phy: 62	Assess the effect of a brief counselling intervention offered by family doctors to women identified through IPV screening	Health and lifestyle survey, World Health Organization Quality of Life (WHOQOL)-Bref instrument*, Short Form-12 (SF-12)*, Hospital Anxiety and Depression Scale (HADS)*	- We suggest that family doctors should be trained to ask about the safety of women and children, and to provide supportive counselling for women experiencing abuse, because our findings suggest that, although we detected no improvement in quality of life, counselling can reduce depressive symptoms<br>- Postal screening might not reach a large proportion of women<br>- Trial does not lend support to screening for IPV in health care settings
Sanci 2015	Australia	Cluster randomised trial	Adults	Pat, Phy, PN	Pat: 620	Pat: 75.7	Evaluate the effectiveness of a complex	HEADSS, Kessler's Scale of emotional distress	- Intervention changed interaction between clinicians

Study	Country	Study type	Age group	Population	Sample size	Female sex, %	Study aim(s)	Name of instrument(s)	Author's conclusions
					Prac: 39	Phy: 51	intervention implementing best practice guidelines recommending clinicians screen and counsel young people across multiple psychosocial risk factors, on clinicians' detection of health risks and patients' risk taking behaviour	(K10)*, Composite Abuse Scale (CAS)*	and young patients: included greater discussion of health risk behaviours and abuse in relationships, which contribute to disease burden, and may have resulted in greater detection of these health risks<br>- High proportion of young people attending PC services are engaging in health risks; with an intervention there are shifts in clinician behaviour and promising indicators that shifts in young people's risk-taking are possible<br>- Complex intervention, compared to a simple educational seminar for practices, improved detection of health risk behaviours in young people, impact on health

Study	Country	Study type	Age group	Population	Sample size	Female sex, %	Study aim(s)	Name of instrument(s)	Author's conclusions
									outcomes was inconclusive
Webb 2015	Australia	Mixed methods (workshops, interviews)	Young people	Pat, Phy, PN	Pat: 16 Par: 8 Phy: 8 PN: 11	Pat: 68.75 Par: 100 Phy: 50 PN: 100	Investigate the design requirements for a health screening tool	HEEADSSS (Home environment, Education and employment, Eating, peer-related Activities, Drugs, Sexuality, Suicide/depression, Safety from injury and violence)	- Overall, young people were enthusiastic about the screening tool and believed it would improve their experience of seeing their GP<br>- A number of key benefits to using a tool were identified, such as that it would increase confidence in and decrease awkwardness of discussing sensitive issues with their GP, enable them to plan what they would say before the consultation, assist them to be more independent and take a more active role in their interaction with their GP<br>- The tool could help identify the most salient health issues

Study	Country	Study type	Age group	Population	Sample size	Female sex, %	Study aim(s)	Name of instrument(s)	Author's conclusions
Blom 2016	Netherlands	Cluster randomised trial	Elderly people	Pat, Phy	Pat: 7,285 Phy: 104	Pat: 57.7	Assess effectiveness and cost-effectiveness of a simple structural monitoring system to detect the deterioration in somatic, functional, mental, or social health of individuals followed by the execution of a care plan	No specific name ('Screening questionnaire')	- GPs felt that new information had emerged from the screening, indicating in particular their possible 'blind spot' for mental and social issues<br>- Some GPs feared that 'medicalisation' was stimulated by the screening<br>- GPs experienced more control over the care situation and were more aware of the functioning and wishes of the older people<br>- However, they found the protocolised way of working difficult and suggested that it was perhaps more suited to the practice nurse<br>- Some GPs preferred to focus on the medical task.<br>- Organising multidisciplinary

Study	Country	Study type	Age group	Population	Sample size	Female sex, %	Study aim(s)	Name of instrument(s)	Author's conclusions
									consultations was found to be cumbersome
Tak 2016	Netherlands	Mixed methods (literature review, evaluation by experts)	Elderly people	Pat	180	66	Develop and validate a short observation list that can be used to detect a broad range of mental disorders and social problems	Observation List for mental disorders and social Problems (OLP)	- The observation list with 14 indicators in five problem areas is preliminary valid, reliable, and can be used during a regular visit<br>- It is short, easy to use (taking only a few minutes to complete), and both GPs and home-care workers indicated that the instrument could be used in daily practice and that it allowed them to observe patients before focusing on specific problems<br>- OLP could objectify the existing gut feeling of the observer and justify further screening
Ambresin 2017	Australia	Cluster randomised	Young people	Pat, Phy, PN	Pat: 901 Phy: 78	Pat: 74.4	Examine whether an intervention also improved	HEADSS, Kessler's Scale of	NR

Study	Country	Study type	Age group	Population	Sample size	Female sex, %	Study aim(s)	Name of instrument(s)	Author’s conclusions
		controlled trial				Phy: 52.6	GPs’ sensitivity in detecting probable mental disorders and examine screening rates for various psychosocial health risks and other psychometric properties of detection accuracy	emotional distress (K10)	
Geyti 2018	Denmark	Prospective study	Adults	Cit	5,970	50.6	Examine mental health care trajectories after mental health screening combined with feedback on both positive and negative screening results to the GP	Short Form Health Survey (SF-12)	- MH screening followed by feedback on both positive and negative screening results to the GP contribute to both initiation and cessation of mental health care<br>- Systematic MH screening may raise GP awareness of the current need for treatment in their listed patients

Study	Country	Study type	Age group	Population	Sample size	Female sex, %	Study aim(s)	Name of instrument(s)	Author's conclusions
Geyti 2020	Denmark	Prospective study	Adults	Cit	350	56.6	Investigate the extent of initiation of mental health care after identification of poor mental health and identify factors associated with non-initiation	Short Form Health Survey (SF-12)	- Systematic provision of mental health test results to GPs may improve the identification of cases in need of mental health care, but does not translate into initiation of mental health care<br>- Much more than screening is required if we are to improve mental health care for adults<br>- New efforts should target groups that are less likely to initiate mental health care, such as men with poor mental health
Klein 2020	USA	Literature / Narrative review	Adolescents, Young people	Pat	N/A	N/A	Reflect the complex biological growth and social role transitions that occur during adolescence	Strengths, school, home, activities, drugs, emotions/eating, sexuality, safety (SSHADESS)	NR

Pat, Patients; Phy, Physicians; Res, Residents; PN, Practice nurses; CMHN, Community mental health nurses; Pract, Practices; Par, Parents; Cit, Citizens; Care, Caregivers; PC, primary care; IPV, intimate partner violence
*Pregnant women; **Veterans
