# Supplementary material for: Identifying patients with psychosocial problems in general practice: A scoping review
Source: Front Med (Lausanne). 2023 Feb 8;9:1010001. doi: 10.3389/fmed.2022.1010001 (PMC9945547; doi:10.3389/fmed.2022.1010001)
Supplement: Supplementary file 5 [file Table_5.docx]

Supplementary Material

**Table 5**. Excluded studies with reasons for exclusion

| Study | Title | Reason for exclusion |
| --- | --- | --- |
| Metcalfe 1978 | The recognition of family and social problems by general practitioners: towards developing a taxonomy | No specific tool described |
| Tulloch 1979 | A randomised controlled trial of geriatric screening and surveillance in general practice | No specific tool described |
| Palsson 1985 | Development of a screening method for probable somatising syndromes | Wrong focus of the tool used |
| Kamerow 1987 | Is screening for mental health problems worthwhile in family practice? An affirmative view | Wrong focus of the tool used |
| Resnicow 1987 | Screening practices of family physicians: a comparison of STFM and AAFP members | Full text not available |
| Giel 1990 | Detection and referral of primary-care patients with mental health problems: the second and third filter | Wrong type of source of evidence |
| Crossley 1992 | Assessment of psychological care in general practice | Wrong concept |
| Gold 1994 | The interview and the social problems | No specific tool described |
| Bensing 1995 | Patient-directed gaze as a tool for discovering and handling psychosocial problems in general practice | No specific tool described |
| Feldman 1995 | Detecting psychological distress among patients attending secondary health care clinics: self-report and physician rating | Wrong setting |
| Olfson 1995 | Recognition of emotional distress in physically healthy primary care patients who perceive poor physical health | No specific tool described |
| Howe 1996 | ‘I know what to do, but it’s not possible to do it’: general practitioners' perceptions of their ability to detect psychological distress | No specific tool described |
| Scicchitano 1996 | Illness behaviour and somatisation in general practice | Wrong population |
| Cape 1999 | Patients’ reasons for not presenting emotional problems in general practice consultations | Wrong concept |
| Robinson 1999 | Counselling by primary care physicians of patients who disclose psychosocial problems | Wrong concept |
| Cape 2000 | Patient-rated therapeutic relationship and outcome in general practitioner treatment of psychological problems | Wrong concept |
| del Piccolo 2000 | Psycho-social problem disclosure during primary care consultations | Full text not available |
| Cape 2001 | How general practice patients with emotional problems presenting with somatic or psychological symptoms explain their improvement | Already included |
| Glascoe 2001 | Increasing identification of psychosocial problems | Wrong type of source of evidence |
| Pfaff 2001 | Training general practitioners to recognise and respond to psychological distress and suicidal ideation in young people | Wrong outcomes |
| Potts 2001 | Lack of mental well-being in 15-year-olds: an undisclosed iceberg? | Wrong population |
| Stirling 2001 | Deprivation, psychological distress, and consultation length in general practice | Wrong concept |
| Jacobson 2002 | Tackling teenage turmoil: primary care recognition and management of mental ill health during adolescence | Wrong type of source of evidence |
| Kates 2002 | Counsellors in primary care: benefits and lessons learned | No specific tool described |
| Rosenberg 2002 | Determinants of the diagnosis of psychological problems by primary care physicians in patients with normal GHQ-28 scores | Wrong concept |
| Sayers 2002 | Early detection of mental health problems in older people | Wrong focus of the tool used |
| Armstrong 2004 | What constructs do GPs use when diagnosing psychological problems? | Wrong concept |
| Huffman 2004 | Early detection of young children’s mental health problems in primary care settings | Wrong population |
| Iliffe 2004 | The development of a short instrument to identify common unmet needs in older people in general practice | Wrong focus of the tool used |
| Maginn 2004 | The detection of psychological problems by general practitioners—influence of ethnicity and other demographic variables | Psychiatric focus |
| Armstrong 2005 | A comparison of GPs and nurses in their approach to psychological disturbance in primary care consultations | Wrong focus of the tool used |
| Romer 2005 | The role of primary care physicians in detection and treatment of adolescent mental health problems | No specific tool described |
| Collins 2006 | Managing depression in primary care: community survey | No specific tool described |
| Brown 2007 | The discussion and identification of youth psychosocial problems during primary care visits | Wrong population |
| Fleming 2007 | The mental health of adolescents—assessment and management | No specific tool described |
| Gunn 2008 | Who is identified when screening for depression is undertaken in general practice? Baseline findings from the Diagnosis, Management and Outcomes of Depression in Primary Care (diamond) longitudinal study | Wrong focus of the tool used |
| Hagan 2008 | Discerning bright futures of electronic health records | Wrong type of source of evidence |
| Horwitz 2008 | Screening for depression in general medical practice: how can natural sadness be distinguished from major depressive disorder? | No specific tool described |
| Walters 2008 | Help-seeking preferences for psychological distress in primary care: effect of current mental state | Wrong concept |
| de la Osa 2009 | Brief mental health screening questionnaire for children and adolescents in primary care settings | Tool based on parental report |
| Brown 2010 | Screening to identify mental health problems in paediatric primary care: considerations for practice | Tool based on parental report |
| Coetzee 2010 | Detecting post-deployment mental health problems in primary care | No specific tool described |
| Feder 2011 | Identification and Referral to Improve Safety (IRIS) of women experiencing domestic violence with a primary care training and support programme: a cluster randomised controlled trial | No specific tool described |
| Harrison 2011 | Adolescent health screening practices by physicians in Jamaica | No specific tool described |
| Hegarty 2011 | Intimate partner violence—identification and response in general practice | No specific tool described |
| Allen 2012 | Mixed methods evaluation research for a mental health screening and referral clinical pathway | Wrong setting |
| Bosmans 2012 | Cost-effectiveness of problem-solving treatment in comparison with usual care for primary care patients with mental health problems: a randomised trial | Already included |
| Hamrin 2012 | Evaluation and management of paediatric and adolescent depression | Psychiatric focus |
| Goodyear-Smith 2013 | The eCHAT program to facilitate healthy changes in New Zealand primary care | Wrong type of source of evidence |
| Klein 2014 | HEEADSSS 3.0: the psychosocial interview for adolescents updated for a new century fueled by media | Wrong setting |
| Bass 2015 | The pocket psychiatrist: tools to enhance psychiatry education in family medicine | Psychiatric focus |
| Lette 2015 | Initiatives on early detection and intervention to proactively identify health and social problems in older people: experiences from the Netherlands | No specific tool described |
| Santo 2017 | Addressing complex patients’ psychosocial priorities during time-limited primary care visits | Full text not available |
| Exner 2018 | Cross-cultural validation of the German version of the Four-Dimensional Symptom Questionnaire (4DSQ) in multimorbid elderly people | Wrong focus of the tool used |
| Gidding 2018 | PsyScan e-tool to support diagnosis and management of psychological problems in general practice: a randomised controlled trial | Wrong population |
| van der Burg 2018 | PsyScan e-tool to support diagnosis and management of psychological problems in general practice | Wrong type of source of evidence |
| Ziadni 2018 | A life-stress, emotional awareness, and expression interview for primary care patients with medically unexplained symptoms: a randomised controlled trial | Wrong population |
| Martel 2019 | YouthCHAT as a primary care e-screening tool for mental healthiIssues among Te Tai Tokerau youth: protocol for a co-design study | Wrong type of source of evidence |
